# *In silico* mechanisms of arsenic trioxide-induced cardiotoxicity

**DOI:** 10.3389/fphys.2022.1004605

**Published:** 2022-12-15

**Authors:** Yacong Li, Runlan Wan, Jun Liu, Weichao Liu, Lei Ma, Henggui Zhang

**Affiliations:** ^1^ Beijing Academy of Artificial Intelligence, Beijing, China; ^2^ Department of Oncology, The Affiliated Hospital of Southwest Medical University, Luzhou, China; ^3^ School of Computer Science and Technology, Harbin Institute of Technology, Harbin, China; ^4^ Key Laboratory of Medical Electrophysiology, Ministry of Education & Medical Electrophysiological Key Laboratory of Sichuan Province, Institute of Cardiovascular Research, Southwest Medical University, Luzhou, China; ^5^ National Biomedical Imaging Center, Peking University, Beijing, China; ^6^ Biological Physics Group, School of Physics and Astronomy, The University of Manchester, Manchester, United Kingdom

**Keywords:** arsenic trioxide, drug cardiotoxicity, ionic channel, cardiac modeling, long QT

## Abstract

It has been found that arsenic trioxide (ATO) is effective in treating acute promyelocytic leukemia (APL). However, long QT syndrome was reported in patients receiving therapy using ATO, which even led to sudden cardiac death. The underlying mechanisms of ATO-induced cardiotoxicity have been investigated in some biological experiments, showing that ATO affects human ether-à-go-go-related gene (hERG) channels, coding rapid delayed rectifier potassium current (*I*_*Kr*_), as well as L-type calcium (*I*_*CaL*_) channels. Nevertheless, the mechanism by which these channel reconstitutions induced the arrhythmia in ventricular tissue remains unsolved. In this study, a mathematical model was developed to simulate the effect of ATO on ventricular electrical excitation at cellular and tissue levels by considering ATO’s effects on *I*_*Kr*_ and *I*_*CaL*_. The ATO-dose-dependent pore block model was incorporated into the *I*_*Kr*_ model, and the enhanced degree of ATO to *I*_*CaL*_ was based on experimental data. Simulation results indicated that ATO extended the action potential duration of three types of ventricular myocytes (VMs), including endocardial cells (ENDO), midmyocardial cells (MCELL), and epicardial cells (EPI), and exacerbated the heterogeneity among them. ATO could also induce alternans in all three kinds of VMs. In a cable model of the intramural ventricular strand, the effects of ATO are reflected in a prolonged QT interval of simulated pseudo-ECG and a wide vulnerable window, thus increasing the possibility of spiral wave formation in ventricular tissue. In addition to showing that ATO prolonged QT, we revealed that the heterogeneity caused by ATO is also an essential hazard factor. Based on this, a pharmacological intervention of ATO toxicity by resveratrol was undertaken. This study provides a further understanding of ATO-induced cardiotoxicity, which may help to improve the treatment for APL patients.

## Introduction

Arsenic trioxide (ATO), a traditional Chinese medicine, has been reported to be used to treat acute promyelocytic leukemia (APL) in 1997 (Chen et al., 1997). After that, scientists discovered that a combination of ATO and all-trans retinoic acid (ATRA) almost cured APL (Shen et al., 2004), whose molecular and cellular mechanisms have also been elucidated (Zhang et al., 2010). In recent years, ATO was screened to rescue the p53 folding function (Chen et al., 2021). In oncology, this is a breakthrough, since the p53 mutation is the most common mutation among cancer patients, which has shown great therapeutic potential but had never been rescued before this research. As a result, ATO is a promising drug in oncotherapy and deserves to be further investigated and applied.

In addition to the efficacy, the safety of ATO also needs to be assessed in clinical trials. It has been reported that ATO may generate cardiotoxicity as well as hepatotoxicity (Mathews et al., 2006; Alexandre et al., 2018). Cardiotoxicity is reflected in the prolonged QT interval of electrocardiograms (ECGs) (Soignet et al., 2001), called long QT syndrome (LQT), which may lead to torsade de pointes tachycardia (TdP) (Unnikrishnan et al., 2001; Hai et al., 2015) and even threaten life (Westervelt et al., 2001; Lenihan and Kowey, 2013). There was also a case of ventricular tachycardia with a normal QT interval in ATO therapy (Ducas et al., 2011), which further warned of the potential arrhythmia risk. A study manifested that the combination of ATRA and ATO therapy can reduce side effects and has less toxicity than ATO treatment alone (Hu et al., 2009). Nevertheless, a Position Paper published by the European Society of Cardiology emphasized that ATO was more related to QT prolongation than other reported anticancer drugs, and it also has a higher chance of causing sudden death due to TdP (Zamorano et al., 2016). Therefore, there is a strong need to further investigate the mechanisms of ATO-induced cardiotoxicity.

Because of the clinical observation of ATO-induced cardiotoxicity, a series of animal studies, including subcellular and cellular experiments, were carried out. According to experimental research, ATO acts on cardiomyocytes (CMs) mainly *via* potassium channels and calcium channels. The human ether-à-go-go-related gene (hERG) codes rapid delayed rectifier potassium current (*I*_*Kr*_) in the human heart, which is susceptible to ATO. Exposure to ATO for 20 min can suppress the hERG channel in hERG-transfected CHO cells (Drolet et al., 2004), whereas an experiment in HEK293 cells (Ficker et al., 2004) indicated that short-term application of ATO did not affect the hERG current and could not alter the action potential duration (APD) in guinea pig ventricular myocytes (VMs). This study revealed that long-term ATO suppressed the *I*_*Kr*_ current in HEK293 cells, and the dosage of ATO directly determined the reduction degree of *I*_*Kr*_ (Ficker et al., 2004). The same phenomenon can also be observed in other HEK293 experiments (Zhao et al., 2015; Yan et al., 2017), and the inhibiting effect of ATO on the hERG channel has also appeared in rodent animals, such as guinea pig VMs (Ficker et al., 2004; Zhao et al., 2014), neonatal rat VMs (NRVMs) (Zhao et al., 2015), and neonatal mouse cardiomyocytes (Liu et al., 2017). In addition, the underlying RNA regulation mechanisms of ATO-impaired hERG were revealed (Shan et al., 2013; Zhao et al., 2015). The effect of ATO on slow delayed rectifier potassium current (I_Ks_) is controversial. The I_Ks_ of CHO cells were susceptible to short-term exposure to ATO (Drolet et al., 2004), but in guinea pig VMs, ATO did not have an apparent influence on I_Ks_ density (Ficker et al., 2004). A similar controversy also appeared in the inward rectifier potassium current (I_K1_). Chronic ATO administration inhibited I_K1_ significantly by reducing Kir2.1 protein expression levels in guinea pig CMs (Chu et al., 2012; Shan et al., 2013) and neonatal rat CMs (Chen et al., 2010; Chu et al., 2012). However, a guinea pig experiment did not show an obvious change in I_K1_ after overnight ATO treatment (Ficker et al., 2004). The drug-delivery method and its dosage should be responsible for this difference between the results. Furthermore, calcium channels are sensitive to ATO. Experiments in guinea pig VMs (Sun et al., 2006) and NRVMs (Chen et al., 2010; Yan et al., 2017) reported an increase in L-type calcium current (*I*_*CaL*_) under the action of ATO at different dosages. The peak of intracellular calcium concentration ([Ca^2+^]_i_) was also markedly increased in the presence of ATO (Yan et al., 2017), while the diastolic [Ca^2+^]_i_ level did not change (Chen et al., 2010). Consistently, it has been verified that ATO can prolong APD in different cell types, including guinea pig VMs (Sun et al., 2006), NRVMs (Chen et al., 2010), HEK293 cells (Ficker et al., 2004), and human-induced pluripotent stem cell-derived cardiomyocytes (hiPS-CMs) (Yan et al., 2017). This finding was consistent with the clinical observation that ATO caused LQT in animal studies (Chen et al., 2010). Research on the potential signaling mechanisms of ATO-induced LQT revealed that ATO promoted the secretion of transforming growth factor-β1 (TGF-β1), which led to fibrosis and inhibited hERG and Kir2.1 protein in CMs, thus causing LQT syndrome (Chu et al., 2012). Although the above experimental results greatly helped understand ATO-induced cardiotoxicity, most studies have been conducted on a single ion channel and have not directly examined using human cardiomyocytes.

It is of great significance in clinical practice to find a way to ameliorate the side effects of ATO. To date, several drugs have been attempted to do this, including antiallergic drugs [such as fexofenadine and astemizole (Yan et al., 2017)], hypoglycemic drugs [such as glibenclamide (Drolet et al., 2004)], cardiovascular drugs [such as nisoldipine (Ficker et al., 2004), ranolazine (Yan et al., 2017) and choline (Sun et al., 2006)], antagonists (Chu et al., 2012) and organic compounds [such as resveratrol (Zhao et al., 2014; Yan et al., 2017), eugenol (Binu et al., 2017) and omega-3 fatty acid (Varghese et al., 2017)]. Fexofenadine can increase the *I*_*Kr*_ of HEK293 cells and shorten APD in both NRVMs and hiPS-CMs treated with 3 μM ATO (Yan et al., 2017). Ranolazine, astemizole and glibenclamide also acted on potassium channel proteins. Ranolazine corrected hERG expression in HEK293 and NRVMs but failed to reverse the damaged hERG channel in hiPS-CMs (Yan et al., 2017). However, astemizole did not have remarkable assuasive effects on the long APD caused by ATO (Yan et al., 2017). Resveratrol (Yan et al., 2017) and choline (Sun et al., 2006) attenuated ATO toxicity by inhibiting the *I*_*CaL*_ channel, and resveratrol exerted a better rescue effect than potassium-intervened agents (Yan et al., 2017). Moreover, lead compound optimization was also reported to be a strategy that alleviated ATO toxicity (Zhou et al., 2016). In addition, the regulatory mechanism of TGF-β1 under ATO treatment (Chu et al., 2012; Liu et al., 2017) provided new methods for preventing hERG and Kir2.1 protein damage by treatment with the protein kinase A (PKA) antagonist H89 and the TβR-I inhibitor LY364947 (Chu et al., 2012).

ATO is a vital agent in the field of clinical oncotherapy. Research is being conducted to unravel and alleviate ATO-induced cardiotoxicity. However, the mechanism of ATO-induced cardiotoxicity is not well understood, especially at the myocardial tissue level. In the present study, we constructed a multiscale mathematical model to simulate cardiac electrical activity in the presence of ATO, by which the generating process of arrhythmia induced by ATO treatment can be delineated from ionic channels to cardiac tissue. In this way, the effect of ATO on single CMs can be extended to a macroscopic level to further predict and analyze its underlying risks. Using the results from this study, we gained a new perspective on ATO-induced cardiotoxicity, such as tissue electrical heterogeneity, vulnerability to arrhythmogenesis and electrical alternans, and provided a method for finding the right dose and a pharmacological rescue scheme for ATO treatment.

## Methods

### Modeling single VMs and the binding interaction between ATO and the *I*_*Kr*_/*I*_*CaL*_ channel

The human VM models, including endocardial cells (ENDO), midmyocardial cells (MCELL) and epicardial cells (EPI), followed ten Tusscher’s model (TNNP06) because of its application in alternans and reentry (ten Tusscher and Panfilov, 2006). The membrane potential of a single VM can be described by the following ordinary differential equation:

$$\frac{dV}{dt}=-\frac{I_{ion}+I_{stim}}{C_{m}}$$ (1)

where *V* is the membrane potential; *t* is time; *I*_*stim*_ is the stimulation current; *C*_*m*_ is the cell capacitance. *I*_*ion*_ is the sum of transmembrane ionic currents, including:

$$I_{ion}=I_{Na}+I_{K1}+I_{to}+I_{Kr}+I_{Ks}+I_{CaL}+I_{NaK}+I_{NaCa}+I_{pCa}+I_{pK}+I_{bCa}+I_{bNa}$$ (2)

Here, we simulated the electrophysiological activities of VMs incubated with ATO by modifying a potassium channel, *I*_*Kr*,_ and a calcium current, *I*_*CaL*_. The formulations of all ionic channel currents can be referenced in the TNNP06 model (ten Tusscher and Panfilov, 2006).

According to a simple pore block theory (Yuan et al., 2014), the binding interaction between ATO and *I*_*Kr*_ can be modeled by a blocking factor λ that denoted the blocking degree of ATO to the maximum conductance of the targeted ion channel. This blocking factor λ can be described by a Hill equation as follows:

$$\lambda =\frac{1}{1+{\left(\frac{D}{IC50}\right)}^{nH}}$$ (3)

where *D* is the concentration of ATO, *IC50* is the ATO concentration at which 50% blockade of the binding site occurs and *nH* is the Hill coefficient. As a result, the remaining maximum conductance index of *I*_*Kr*_ is:

k=1−λ (4)

Consequently, the formulation of *I*_*Kr*_ can be described as follows:

$$I_{Kr}=k\cdot G_{Kr}\sqrt{\frac{K_{o}}{5.4}}x_{r1}x_{r2}\left(V-E_{K}\right)$$ (5)

where *G*_*Kr*_ is the conductance of *I*_*Kr*_, K_o_ is the extracellular K^+^ concentration, *x*_*r1*_ is an activation gate, *x*_*r2*_ is an inactivation gate, and *E*_*K*_ is the reversal potential.

**FIGURE 1:**
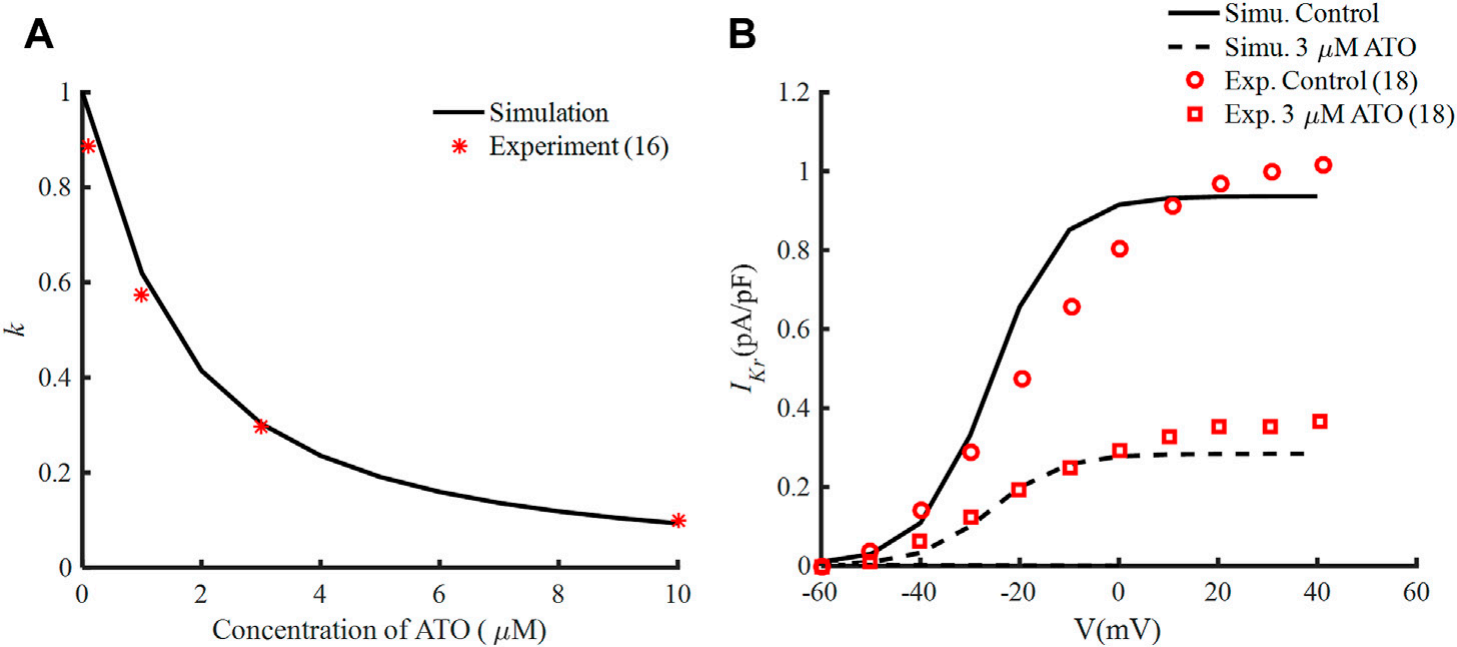
Effects of ATO and the hERG channel current. **(A)** The normalized remaining maximum conductance of *I*_*Kr*_ blocked by ATO. **(B)** Tail current of *I*_*Kr*_ under the conditions of control and 3 μM ATO in the simulation model (black lines) and an experiment (red circle and square), respectively.

According to the experimental data (Ficker et al., 2004), *IC50* was 1.5 μM and *nH* was fitted at −1.2, so the interaction between ATO concentration and k is demonstrated in Figure 1A. The tail currents of *I*_*Kr*_ under control and 3 μM ATO in the present simulation model and experimental measurement (Yan et al., 2017) are shown in Figure 1B. Our model could well fit the experimental data, which indicated the accuracy of the present model.

Experiments indicated that exposure to 3 μM ATO for 24 h tripled the density of *I*_*CaL*_ in guinea pig VMs (Ficker et al., 2004) or doubled it in NRVMs (Yan et al., 2017). Another animal experiment showed that when ATO administration led to a double *I*_*CaL*_ density, the activation curve of *I*_*CaL*_ was shifted negatively (Chen et al., 2010).

In the present model, the formulation of *I*_*CaL*_ was as follows:

$$I_{CaL}=\theta \cdot G_{CaL}dff_{2}f_{CaSS}4\frac{\left(V-15\right)F^{2}}{RT}\frac{0.25Ca_{SS}e^{2\left(V-15\right)F/RT}-Ca_{o}}{e^{2\left(V-15\right)F/RT}-1}$$ (6)

where *d* is a voltage-dependent activation gate:

$$d_{\infty }=\frac{1}{1+{e^{\left(V_{1/2}-V\right)}}^{/7.5}}$$ (7)

Two parameters that were modified to simulate the effect of ATO on *I*_*CaL*_ were θ and *V*_*1/2*_, which represented the change in the conductance of *I*_*CaL*_ (*G*_*CaL*_) and the half activation voltage (*V*_*1/2*_) of the activation gate d) respectively. They were estimated according to the above biological experimental results, and the corresponding values are listed in Table 1. The meanings of the other parameters in Eq. (6) can be seen in Ref. (ten Tusscher and Panfilov, 2006).

**TABLE 1:** The effect of ATO on *I*_*CaL*_.

Concentration of ATO (μM)	θ (index of G_CaL_)	V_1/2_ of the activation gate d (mV)
0	0	−8
1	33	−8
2	66	−8
3	100	−10

Experiments observed that resveratrol can suppress the *I*_*CaL*_ channel current, thus shortening APD (Zhang et al., 2006), and it was shown to increase the *I*_*Kr*_ channel current (Zhao et al., 2014). Consequently, it was suitable to alleviate cardiotoxicity caused by ATO (Zhao et al., 2014; Yan et al., 2017). The effect of resveratrol on the VMs treated with 3 µM ATO was simulated by manipulating the conductance of *I*_*CaL*_ and *I*_*Kr*_. According to experimental data, under the condition of 3 µM ATO, 10 µM resveratrol decreased the *I*_*CaL*_ density from twice the original value to approximately 1.3 times (Yan et al., 2017). And 10 µM resveratrol recovered *I*_*Kr*_ by approximately 33% of its density under the impact of 3 µM ATO (Zhao et al., 2014). The same rescue ratio of resveratrol was applied in the present model; thus, the formulation of *I*_*Kr*_ and *I*_*CaL*_ was changed as follows:

$$I_{Kr}=n_{Res}\cdot k\cdot G_{Kr}\sqrt{\frac{K_{o}}{5.4}}x_{r1}x_{r2}\left(V-E_{K}\right)$$ (8)

$$I_{CaL}=m_{Res}\cdot \theta \cdot G_{CaL}dff_{2}f_{CaSS}4\frac{\left(V-15\right)F^{2}}{RT}\frac{0.25Ca_{SS}e^{2\left(V-15\right)F/RT}-Ca_{o}}{e^{2\left(V-15\right)F/RT}-1}$$ (9)

where *n*_*Res*_ and *m*_*Res*_ are the coefficients of resveratrol’s effect on *I*_*Kr*_ and *I*_*CaL*_, whose values were 1.33 and 1.3, respectively, under 10 µM resveratrol.

To assess the degree of pharmacological rescue, we defined the rescue ratio as follows:

$$r=1-\frac{APD_{90Drug+ATO}-APD_{90Control}}{APD_{90ATO}-APD_{90Control}}$$ (10)

In which APD_90_ means time duration from depolarization to 90% repolarization, $APD_{90Control}$ is the APD_90_ of original VM cells with 0 µM ATO, $APD_{90ATO}$ is the APD_90_ with 3 µM ATO, and $APD_{90Drug+ATO}$ is the APD_90_ with 3 µM ATO as well as drug. The greater *r* is, the better the therapeutic effect of the drug. Particularly, when *r* is equal to 0, the drug does not work. When *r* is equal to 1, the side effect of ATO is completely rescued.

Eq. (1) was solved by the forward Euler method with a time step of 0.02 m. The single VM model was pulsed under stimulus currents of −52 pA/pF with a basic cycle length (BCL) of 800 m. The S1-S2 standard protocol was used to depict the restitution curve of a single cell. Ten S1 stimulation currents were applied under a BCL of 800 m, following an S2 stimulation current after a dynamic shortening period. This period was called the S1-S2 interval, and the corresponding APD_90_ of the last cycle was calculated. A dynamic protocol was used to estimate the risk of alternans of VMs, which was conducted by a series of S1 with tapering BCL and corresponding APD_90_ being calculated.

### Modeling ventricular tissue

Non-linear cable theory was applied to build a monodomain ventricular tissue model. As such, the electrical activity of ventricular tissue can be described by a partial differential equation as follows:

$$\frac{\partial V}{\partial t}=\nabla \cdot D\nabla V-\frac{I_{ion}+I_{stim}}{C_{m}}$$ (11)

where ∇ is the spatial gradient operator, $\nabla =\frac{\partial V}{\partial x}$ in the ventricular cable model and $\nabla =\frac{\partial V}{\partial x}+\frac{\partial V}{\partial y}$ in the ventricular tissue model; *V* is the membrane potential; *t* is time; *D* is the diffusion coefficient; *I*_*ion*_ is the sum of transmembrane ionic currents; *C*_*m*_ is the cell capacitance.

Eq. (11) was solved by the finite difference method with a time step of 0.02 m and a space step of 0.25 mm. The D was set at 0.08 mm^2^/ms (Luo et al., 2017) so that the conductivity velocity of electrical waves in ventricular tissue was 0.7 m/s, which was consistent with experimental observations (Taggart et al., 2000).

ECG is used to describe the body surface potential in the clinic. It can be estimated according to cellular electrophysiological processes (Gima and Rudy, 2002). Here, we calculated a pseudo-ECG by the following equation:

$$\Phi =\int \frac{k\nabla V\cdot \vec{r}}{r^{3}}dV$$ (12)

where *k* is a constant; *V* is the membrane potential; $\vec{r}$ is a vector from any point in tissue to the electrode; *r* is the length of $\vec{r}$ .

S1-S2 stimuli were used to evaluate the vulnerability of ventricular cables and tissues *via* the genesis of unidirectional conduction block. In the ventricular cable, S1 stimuli were applied at first five ENDO cells, and S2 stimuli were applied at five EPI cells. Different locations of EPI that applied S1 stimuli were from the site that neighboring MCELLs to the site that was far away from 5 MCELLs, i.e., the 61–65th cells to 65–69th cells. In ventricular tissue, S1 stimuli were also located at ENDO cells, while S2 stimuli were applied at a block of epicardial tissue whose width was less than the whole cardiac width so that spiral waves could be motivated.

The dynamic protocol was conducted in a heterogeneous ventricular cable, in which the S1 stimuli were applied at the first five ENDO cells with a variable cycle length from 300–500 m.

## Results

### Effects of ATO on the action potential of VMs

**FIGURE 2:**
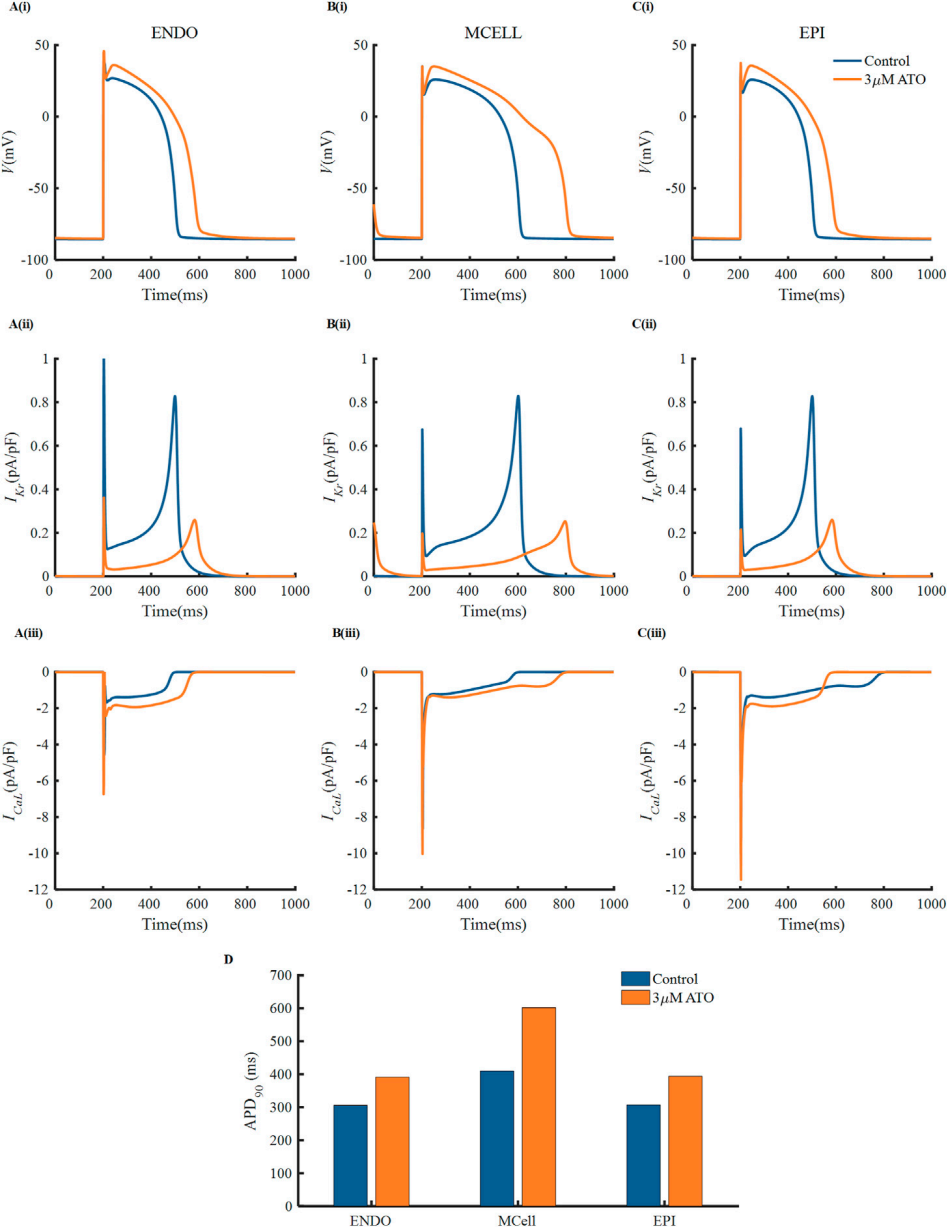
The effect of ATO on the action potentials of different ventricular myocytes. **(A–C)** The membrane potential, (i) (*V*), *I*_*Kr*_ (ii) and *I*_*CaL*_ (iii) of endocardial cells (ENDO), middle cells (MCELL) and epicardial cells (EPI) on the condition of control and 3 μM ATO. **(D)** The APD_90_ of the action potentials in Figure A.

There were experimental data of VMs incubated with 3 µM ATO (Ficker et al., 2004; Yan et al., 2017), in which the subcellular effects of ATO on *I*_*Kr*_ and *I*_*CaL*_ were provided. As a result, we simulated the electrophysiology of three types of VMs in the presence of 3 μM ATO and exerted different stimulation protocols to investigate the change in single-cell membrane potential under ATO intervention. First, a series of periodic stimulation protocol with a BCL of 800 m was conducted, whose corresponding heartbeat was 75 times per minute. According to Eq. (3), 3 μM ATO inhibited the conductance of *I*_*Kr*_ by 70%, thus suppressing *I*_*Kr*_ density in ENDO, MCELL and EPI (Figure 2A(ii)–C(ii)). The administration of 3 μM ATO also doubled the conductance of *I*_*CaL*_ and shifted the activation curve, thus increasing *I*_*CaL*_ density (Figure 2A(iii)–C(iii)). The increased *I*_*CaL*_ also accumulated the intracellular calcium concentration (*[Ca* ^*2+*^ *]*_*i*_) *via* the calcium dynamics in VMs, thus increasing the Na^+^/Ca^2+^ exchanger current (*I*_*NaCa*_) as shown in Supplementary Figure S1. As expected, the decrease in *I*_*Kr*_ and increase in *I*_*CaL*_ and *I*_*NaCa*_ prolonged the APD of VMs to different degrees. ATO increased the APD_90_ of ENDO from 306 m to 391 m, that of MCELL from 410 m to 602 m, and that of EPI from 307 m to 394 m. The increase ratio of APD_90_ were approximately 27.8%, 46.8%, and 28.3%, respectively, in the three types of VMs. It was noteworthy that the APD_90_ increase ratio showed a great difference among different VMs, which may increase the risk of arrhythmia in ventricular tissue. Specifically, the difference in the action potential properties between two adjacent VMs can extend the time window that one kind of cell was in the resting state and the other was in the refractory period, which may lead to unidirectional conduction block, thus producing reentry in cardiac tissue. This will be further analyzed in the following section.

**FIGURE 3:**
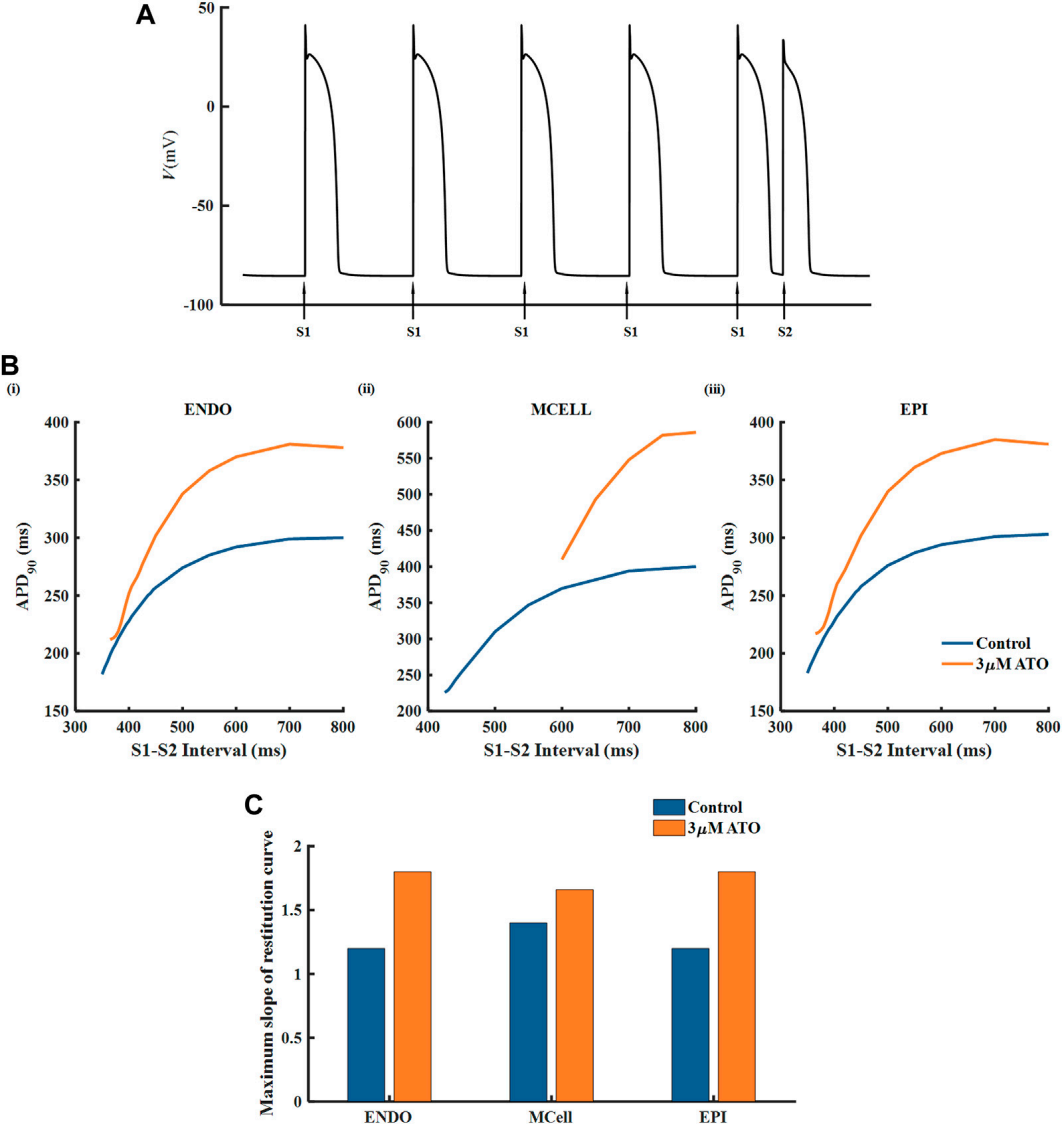
The restitution curves of ventricular myocytes. **(A)** The S1-S2 stimulation protocol. **(B)** The restitution curves of endocardial cells (ENDO), middle cells (MCELL) and epicardial cells (EPI) on the condition of control and 3 μM ATO. **(C)** The maximum slope of restitution curves in Figure **(B)**.

The APD restitution curve of a single cell was deemed to relate to the dynamical behavious of spiral waves in cardiac tissue. Here, we drew an APD restitution curve *via* the S1-S2 stimulation protocol, as shown in Figure 3A. Ten uniform S1 stimuli with a BCL of 800 m were applied before the S2 stimulus (only five S1 stimuli are shown in Figure 3A). With the reduction of the S1-S2 interval, the APD_90_ of the action potential triggered by the S2 stimulus declined until the S2 stimulus could not ignite depolarizing activity. The relationship between the S1-S2 interval and the corresponding APD_90_ formed the restitution curve as shown in Figure 3B. The restitution curve was shifted rightward slightly in ENDO and EPI and dramatically in MCELL, implying that the ATO-incubated VMs cannot support high-frequency pacing activity. The slope of the restitution curve reflects the stability of spiral waves. Results showed that ATO steepened the restitution curves of all kinds of VMs to varying degrees, whose slope is shown in Figure 3C, indicating unstable electrical activities.

**FIGURE 4:**
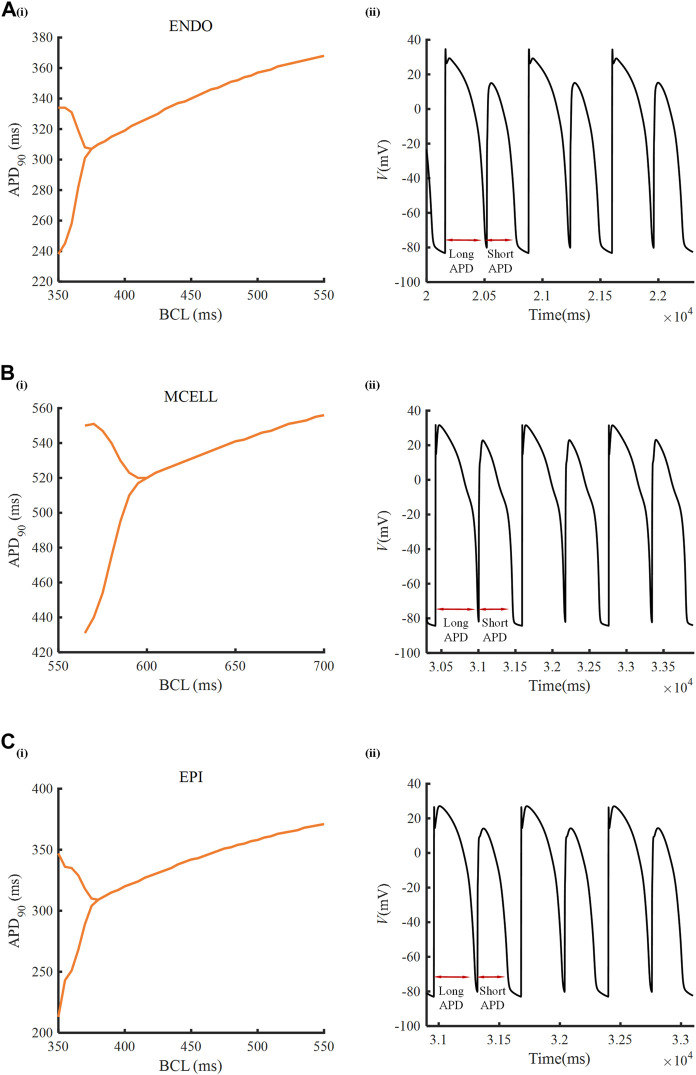
Alternans induced by ATO. **[A(i)–C(i)]** APD_90_ rate-dependent curves of endocardial cells (ENDO), middle cells (MCELL) and epicardial cells (EPI) incubated with 3 μM ATO. **[A(ii)–C(ii)]** The representative membrane potentials of Figure **[A(i)–C(i)]** with the basic cycle lengths at 360, 585, and 360 ms, respectively.

To inspect the electrical activity of ATO-induced VMs under the high-frequency stimulus, a dynamic stimulation protocol was executed by gradually shortening the BCL. In the normal VM model, APD_90_ was unchanged under a specific BCL no matter how small the BCL was (results not shown). In the presence of 3 μM ATO, the APD_90_ in two consecutive beats may be different when BCL was reduced to a threshold. For example, when the BCL was 360 m, the membrane potential of ENDO had two alternans APD_90_ with a long APD_90_ at 334 m and a short APD_90_ at 238 m (Figure 4A(ii)). This kind of periodic APD_90_ change in a fixed BCL is called alternans. Figure 4 indicates that the alternans occurred during a BCL of 350–375 m in ENDO, that of 550–600 m in MCELL and that of 350–380 m in EPI, whose corresponding representative membrane potentials are given in Figure 4A(ii)–C(ii).

### Effects of ATO on the vulnerability of heterogeneous ventricular cables

**FIGURE 5:**
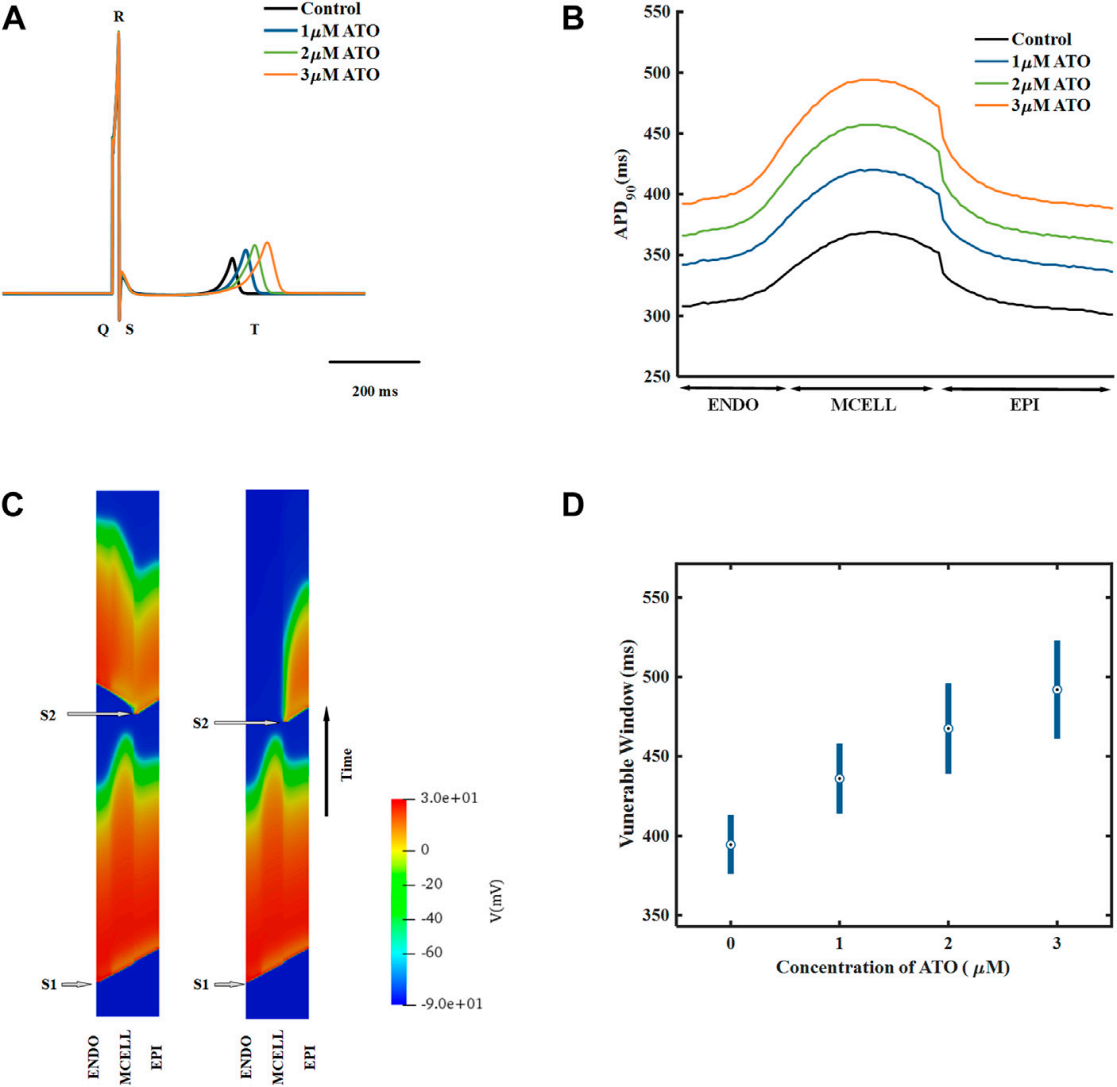
The effect of ATO on the heterogeneous ventricular cable. **(A)** ECG of ventricular cables with varying ATO concentrations. **(B)** Spatial distribution of APD_90_ across the ventricular cable with varying ATO concentrations. **(C)** Space-time plot of normal and unidirectional conduction. **(D)** Vulnerable windows of ventricular cables under varying ATO concentrations.

A heterogeneous ventricular cable including ENDO, MCELL and EPI with a ratio of 25:35:40 (Luo et al., 2017) was designed, and its electrical activities were simulated by solving Eq. (11). According to the membrane potential of the ventricular cable, the pseudo ECG, whose virtual electrode was placed 2.0 cm away from the last EPI, was calculated by Eq. (12). The ECG under different ATO concentrations is shown in Figure 5A. With the increase in ATO concentration, the QT interval rose from 362 m at 0 μM ATO to 477 m at 3 μM ATO, and the amplitude of the T wave slightly increased.

The dispersion of APD in the ventricular cable directly influenced the vulnerability of the cable. Compared with the isolated single cell, the gap junction between VMs decreased the difference in membrane potential duration between cells. Nevertheless, there was still an obvious difference in APD_90_ in space, particularly between MCELL and EPI, as shown in Figure 5B. The incorporation of ATO exacerbated the dispersion. The maximum gap of APD_90_ between two adjacent cells was 17 m in the control and 26 m on the condition of 3 μM ATO (Figure 5B). An S1-S2 stimulation protocol was applied to heterogeneous ventricular cables to detect vulnerability under varying ATO concentrations. Under the condition of long S1-S2 intervals, S2 inspired an electrical wave that can propagate into both MCELL and EPI tissue, as shown in the left panel in Figure 5C. However, in a short S1-S2 interval, the S2-inspired wave can only propagate into EPI tissue because the MCELL was in the refractory period and could not depolarize, as shown in the right panel of Figure 5C. This kind of unidirectional conduction may lead to the formation of reentry in ventricular tissue. As a result, the S1-S2 interval that led to unidirectional conduction was measured to evaluate the underlying arrhythmia risk, which was called the vulnerable window. Figure 5D presents the results. With the increment of ATO dosage, the vulnerable window expanded from 37 m at 0 μM ATO to 62 m at 3 μM ATO, demonstrating an increasing possibility of reentry at the tissue level. The value of the vulnerable window also increased from 376–413 m at 0 μM ATO to 461–532 m at 3 μM ATO. This was because extra ATO prolonged the APD in single VM cells; thus, the refractory period extended. As a result, the S1-S2 interval with a unidirectional conduction block was greater, which was reflected in the increase in the value of the vulnerable window. In addition, S2 was exerted at more locations as described in the Method section. The results at all locations had a coincident density of VM as shown in Table 2.

**TABLE 2:** Effect of ATO on VM in the ventricular cable model.

Concentration of ATO (μM)	S2 location (cell number)	Unidirectional conduction timing range (ms)	Vulnerable window (ms)	Average vulnerable window (ms)
0	61–65	376–413	37	35.6
	62–66	376–415	39	
	63–67	377–414	37	
	64–68	379–413	34	
	65–69	381–412	31	
1	61–65	414–458	44	38
	62–66	414–456	42	
	63–67	416–455	39	
	64–68	415–454	39	
	65–69	416–454	38	
2	61–65	439–496	57	53
	62–66	438–490	52	
	63–67	437–490	53	
	64–68	437–489	52	
	65–69	441–494	53	
3	61–65	461–523	62	70
	62–66	458–524	66	
	63–67	455–523	68	
	64–68	453–522	69	
	65–69	451–521	70	

The results of the dynamic protocol indicated that discordant alternans can be induced in a heterogeneous ventricular cable. A representative result with a BCL of 410 m is shown in Supplementary Figure S2. The ENDO cells presented alternans APD, while some of the short APD was blocked by the MCELL because of its long refractory period.

### Effects of ATO on the vulnerability of heterogeneous ventricular tissue

**FIGURE 6:**
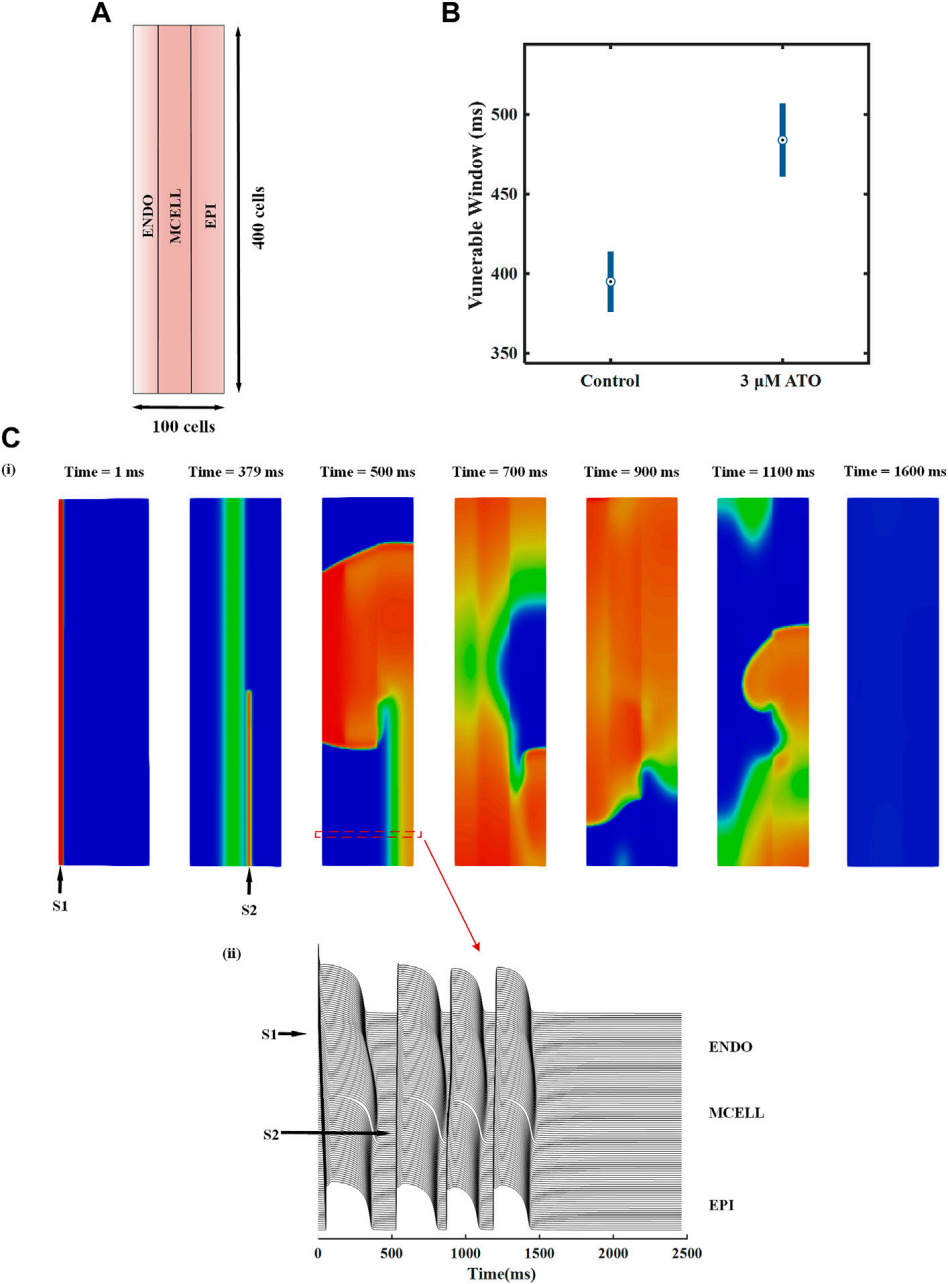
The effect of ATO on heterogeneous ventricular tissue. **(A)** The structure of heterogeneous ventricular tissue. **(B)** The vulnerable window of ventricular tissue in control and 3 μM ATO conditions. **(C)** Snapshots of reentrant excitation waves in the control condition with S1-S2 intervals at 378 m and sequence diagram of the membrane potential of horizontal continuous ventricular myocytes.

We designed a heterogeneous ventricular tissue with a size of 100 × 400 cells (Figure 6A). The length of the tissue included 100 heterogeneous VMs with an ENDO:MCELL:EPI ratio of 25:35:40. Electrophysiology activity with time can be solved by Eq. (11). An essential evaluation index in the two-dimensional (2D) ventricular tissue was reentry, i.e., spiral wave. It was a curved wavefront generated due to unidirectional conduction in tissue. We induced spiral waves through an S1-S2 stimulation protocol in ventricular tissue. A case in the control condition is shown in Figure 6C and the complete videos were attached in the supplementary materials. A case of reentry under the 3 μM ATO condition is also shown in the supplementary materials. An S1 stimulus was applied to the peripheral ENDO tissue with a length of five cells and a width of 400 cells (whole tissue width) to induce a plane wave (the first panel in Figure 6C(i)). During the refractory period of MCELL, an S2 stimulus was applied to the EPI cells that neighbored MCELL, whose width was less than that of the whole tissue (the second panel in Figure 6C(i)). With a specific S1-S2 interval and sufficient S2 stimulus width, a spiral wave can be provoked. The S1-S2 interval in Figure 6C(i) were 378 m. The membrane potentials of horizontal cells in 2D tissue are shown in Figure 6C(ii)), in which a single S2 stimulus could provoke more than one depolarizing potential. In the control condition (the ATO concentration was 0 μM), the S1-S2 interval that can induce spiral waves was from 376 m to 414 m, with a time window of 38 m. When the ATO concentration increased to 3 μM, reentry occurred during the S1-S2 interval of 461–507 m, whose vulnerable window rose to 46 m (Figure 6B).

### Effects of resveratrol on ATO-induced cardiotoxicity

When treated with 3 μM ATO in VMs, 10 μM resveratrol can surpress the excessive *I*_*CaL*_ conductance from twice to 1.3 times (Yan et al., 2017) and increase the remaining maximum conductance index k of *I*_*Kr*_ (Eq. (4)) to 1.3 times (Zhao et al., 2014). The membrane potential of three types of VMs in the presence of 3 μM ATO and 10 μM resveratrol were simulated. The results showed that resveratrol shortened the APD_90_ from 391 to 357 m in ENDO cells, from 602 to 511 m in MCELL cells and from 394 to 360 m in EPI cells. Resveratrol also narrowed the maximum gap of APD_90_ between the three types of VMs from 211 to 154 m. The action potentials of different types of VMs treated with ATO and resveratrol are shown in Supplementary Figure S3.

The effect of resveratrol was further predicted in a ventricular cable model. The S1-S2 stimulation protocol was conducted with 3 μM ATO and 10 μM resveratrol. Simulation results showed that the time window that produced unidirectional conduction was narrowed from 62 m to 45 m under the action of resveratrol. The vulnerable window in different conditions is presented in Table 3.

**TABLE 3:** Effect of resveratrol on the ATO-induced ventricular cable model.

Condition	Unidirectional conduction range of stmulus timings (ms)	Vulnerable window (ms)
Control	376–413	37
3 μM ATO	461–523	62
3 μM ATO + 10 μM resveratrol	435–480	45

## Discussion

### Summary of major findings

As has been widely reported, ATO may cause severe cardiotoxicity when applied to treat APL (Haybar et al., 2019) by interfering with hERG channels (Zhao et al., 2015) as well as *I*_*CaL*_ channels (Chen et al., 2010). Even so, ATO remains one of the most effective drugs for rescuing cancer patients since it has a high long-term survival rate in newly diagnosed APL patients (Hu et al., 2009). As a result, the mechanisms of ATO-induced cardiotoxicity need to be elucidated, based on which more methods can be developed to improve the safety of ATO therapy. The *in silico* method provided an efficient approach to do this. Some mature models have been widely used in drug screening, such as the CiPA model (Park et al., 2019; Han et al., 2020; Ridder et al., 2020; Strauss et al., 2021) and a virtual heart model (Yuan et al., 2014). In this study, a mathematical model was built to delineate the cardiotoxicity of the human ventricle implicated in ATO by modulating the *I*_*Kr*_ and *I*_*CaL*_ channels according to corresponding patch clamp data (Ficker et al., 2004; Chen et al., 2010; Yan et al., 2017). Based on the constructed model, we explored the process of ATO-induced arrhythmia from the subcellular level to the tissue level. The long APD in cells and LQT in cables were triggered by ATO-induced inhibition of *I*_*Kr*_ and facilitation of the *I*_*CaL*_ channel, coinciding with clinical ECG diagnosis (Soignet et al., 2001). Beyond ATO’s explicit side-effect on LQT, it was first uncovered that ATO could augment the heterogeneity between different types of ventricular tissue, which was also an essential predisposing factor of tachycardia. In addition, ATO may induce alternans in all three types of VMs, which indicated an increase in the arrhythmia risk. Moreover, the vulnerability of ventricular tissue increased under the action of ATO, which was a direct factor of cardiac arrhythmia. Despite the underlying detrimental effects of ATO on the human heart, this study demonstrated a potential pharmacological remedy by resveratrol, which is expected to be beneficial for the safety of ATO therapy and provided better prognosis for newly diagnosed APL patients.

### ATO-induced cardiotoxicity

In clinical trials, APL patients who received ATO had a higher risk of suffering from LQT syndrome and even sudden cardiac death (Westervelt et al., 2001). Biologists tried to explain the underlying ionic mechanisms of ATO-induced arrhythmia and found that K^+^ channels (including *I*_*Kr*_, *I*_*Ks*_ and *I*_*K1*_) (Ficker et al., 2004) and Ca^2+^ dynamics (including *I*_*CaL*_ and *[Ca* ^*2+*^ *]*_*i*_) (Chen et al., 2010) might be responsible for this. The effect of ATO on *I*_*K1*_ and *I*_*Ks*_ is controversial. An experiment in CHO cells claimed that ATO could inhibit the *I*_*Ks*_ channel (Drolet et al., 2004). ATO can also impair the expression of Kir2.1 in guinea pigs (Shan et al., 2013) and reduce the *I*_*K1*_ density in guinea pig VMs (Shan et al., 2013) and rat VMs (Chen et al., 2010). However, another study announced that the *I*_*Ks*_ and *I*_*K1*_ of guinea pig VMs had no obvious changes with overnight ATO incubation (Ficker et al., 2004). There were unified results of the inhibitory effect of ATO on *I*_*Kr*_ and its promotive effect on *I*_*CaL*_, whose experimental data were more abundant. As a result, we mainly focused on ATO’s effect on the ventricle *via I*_*Kr*_ and *I*_*CaL*_ channels.

The relationship between several ATO dosages and the degree of inhibition of *I*_*Kr*_ was given in an experiment (Ficker et al., 2004), which provided basic data to depict the binding interaction between ATO and *I*_*Kr*_ in the present model. The I-V curve data of *I*_*CaL*_ were usually measured in the condition of 3 µM ATO. Experiments displayed that long-term exposure to ATO facilitated *I*_*CaL*_ density to approximately 2–3 times and negatively shifted the *V*_*1/2*_ of the activation curve (Ficker et al., 2004; Chen et al., 2010; Yan et al., 2017). According to this, we estimated the subcellular effect of ATO on human VMs and simulated the electrical activity of ATO-disrupted ventricular single cells, cables, and tissue. The simulation result implied a high risk of arrhythmia due to the steep restitution curve and electrical alternans in single VMs. Additionally, beyond the biological experiments, we found that ATO-reconstructed ionic channels not only prolonged the action potential of ventricular cells, but also aggravated the heterogeneity between the three types of VMs. On the one hand, in the present simulation, the QT interval of heterogeneous ventricular cable was 362 m in control and treated with 3 μM ATO prolonged QT interval to 477 m. Clinically, the normal QT interval should range from 350–420 m, over 460 m in women and 440 m in men can be diagnosed with LQT (Vadavanath et al., 2019). Particularly, when QT is more than 500 m, the risk of TdP increases dramatically (Zamorano et al., 2016). This means that the present simulation result with 3 μM ATO was in a hazardous range of QT intervals, which was coincident with clinical manifestations and thus reliable. In addition, simulation studies also reported the underlying risk of long APD. A study indicated that the prolonged APD impaired the repolarization of action potential, so in a short cycle length, the early afterdepolarizations may be evoked (Bai et al., 2017). Another study also proved that the adaptability of ventricular tissue was impaired because of the prolonged APD (Bi et al., 2022). On the other hand, increased ventricular heterogeneity among single VM cells can result in a further detriment in the ventricle, raising the possibility of reentry within the heart. Both factors led to a wide vulnerable window in ventricular tissue, indicating a higher risk of arrhythmia in the heart.

An uncharacteristic discordant alternans can be observed under the action of ATO (Supplementary Figure S2). The tissue alternans in this study were not as obvious as those in heart failure-associated atrial alternans research (Zhao et al., 2020) because except for single cell characters, the decreased CV in atrial tissue was also an essential factor in inducing tissue alternans. There is no evidence that ATO reduced the CV in ventricular tissue, so the alternans did not easily occur in this present simulation.

The ATO concentration in plasma reached 0.34–2 µM with intravenous treatment for APL and acute myeloid leukemia (AML) patients at a dosage of 10 mg/day (Siu et al., 2006). As a result, although there were animal experimental data with ATO concentrations of 0.1–50 µM (Ficker et al., 2004; Sun et al., 2006), we mainly adopted and modeled the data within 0.1–3 µM ATO. As a result, the present model was clinically valuable, based on which ATO-induced arrhythmogenesis can be investigated.

### Pharmacological rescue of ATO toxicity

Despite the possibility of cardiac arrhythmia after administering ATO in both experiments and the simulation, it was still crucial for APL patients. Consequently, drug combinations have been proposed as a means of reducing cardiotoxicity resulting from ATO. Various kinds of drugs have been investigated to attenuate ATO-induced toxicity, but only a few have shown remarkable rescue properties. Fexofenadine is an antiallergic agent. Experiments have shown that 1 μM fexofenadine can increase *I*_*Kr*_ from 30% of the original density to approximately 60% in HEK293 cells incubated with 3 µM ATO (Yan et al., 2017). It was verified to shorten the APD_90_ from 396.43 ± 25.33 (with 3 µM ATO) to 233.30 ± 18.75 m in NRVMs and from 1164.71 ± 40.25 to 942.86 ± 103.11 m in hiPS-CMs (Yan et al., 2017). Nisoldipine, a hypotensive drug, could shorten the APD_90_ of guinea pig VMs with 3 µM ATO treatment from 880 ± 61 to 686 ± 36 m (Ficker et al., 2004). It is known to be the *I*_*CaL*_ blocker, but the quantitative relation between Nisoldipine concentration and *I*_*CaL*_ properties was not given on the condition of ATO. Resveratrol is a natural antioxidation ingredient that can protect the cardiovascular system (Sulaiman et al., 2010; Dudka et al., 2012) by ameliorating structural abnormalities and oxidative damage (Zhao et al., 2008). It can act on both the *I*_*Kr*_ (Zhao et al., 2014) and *I*_*CaL*_ (Yan et al., 2017) channels and shorten the APD_90_ of ATO-incubated cells from 948.3 ± 63.7 m to 522.6 ± 26.3 m in guinea pig VMs (Zhao et al., 2014) and from 1164.71 ± 40.25 to 942.86 ± 103.11 m in hiPS-CMs (Yan et al., 2017). It can be found that resveratrol performed the best rescue effect among all three drugs that had significant protective effects on ATO-incubated CMs, and the rescue ratio was less in hiPS-CMs than in rodent VMs. The data on drug rescue in ATO-incubated CMs are summarized in Table 4.

**TABLE 4:** Effect of drugs on the APD of cardiomyocytes incubated with 3 μM ATO.

Drugs	Subject	Working channel	Control APD_90_ (ms)	ATO APD_90_ (ms)	Drug + ATO APD_90_ (ms)	r
1 μM fexofenadine (Yan et al., 2017)	HEK293	*I*_*Kr*_	N/A	N/A	N/A	N/A
	NRVMs	N/A	171.25 ± 10.58	396.43 ± 25.33	233.30 ± 18.75	72.44%
	hiPS-CMs	N/A	706.98 ± 23.71	1164.71 ± 40.25	942.86 ± 103.11	48.47%
25 nM nisoldipine (Ficker et al., 2004)	Guinea pig VMs	*I*_*CaL*_	495 ± 40	880 ± 61	686 ± 36	50.39%
10 μM resveratrol	hiPS-CMs (Yan et al., 2017)	*I*_*CaL*_	706.98 ± 23.71	1164.71 ± 40.25	942.86 ± 103.11	48.47%
	Guinea pig VMs (Zhao et al., 2014)	*I*_*Kr*_	429.1 ± 26.5	948.3 ± 63.7	522.6 ± 26.3	81.99%
10 μM resveratrol simulation	ENDO	*I*_*CaL*_& *I*_*Kr*_	306	391	357	40.00%
	MCELL	*I*_*CaL*_& *I*_*Kr*_	410	602	511	47.40%
	EPI	*I*_*CaL*_& *I*_*Kr*_	307	394	360	39.08%

To further predict the effect of resveratrol on human VMs incubated with ATO, we simulated ATO/resveratrol-incorporated ventricular models by modifying the *I*_*Kr*_ and *I*_*CaL*_ channels. Although the rescue ratio of resveratrol declined in human VMs, it still remarkably ameliorated ATO cardiotoxicity (Table 4). In the present model, resveratrol not only shortened the APD_90_ of ENDO, MCELL and EPI but also narrowed the difference in APD_90_ between VMs, thus decreasing the vulnerable window of the ventricular cable (Table 3). This study verified that resveratrol has the potential to be applied in clinics to protect the cardiovascular system in ATO-treated patients.

### Limitations

Except for the intrinsic limitations of the basic TNNP06 model (ten Tusscher and Panfilov, 2006), the pharmacological model in this study was not completely accurate due to a lack of abundant experimental data. The patch clamp experiment was performed on HEK293 (Yan et al., 2017) or rodent VMs (Chen et al., 2010), so it was only a prediction of the binding interaction between ATO and human VMs. In another human VM model (O'Hara et al., 2011), the *I*_*Kr*_ channel model was built by the Markov chain model, and the corresponding drug model was extended by incorporating two drug states (Whittaker et al., 2017). In this study, the simple pore block theory was used to depict the effect of ATO on the *I*_*Kr*_ channel because it was suitable for the *I*_*Kr*_ model of TNNP06.

Experiments reported that ATO had an underlying effect on calcium homeostasis by upregulating the expression of CaMKII, which finally caused abnormal CM contraction (Zhang et al., 2016; Zhang et al., 2017). The change in calcium dynamics caused by extra ATO is worth discussing *in silico* after more specific ionic data are obtained.

ATO may also have an impact on other ionic currents, such as I_K1_ (Chen et al., 2010), I_Ks_ (Drolet et al., 2004) and I_Na_ (Ficker et al., 2004), but the literature shows that their effect is not obvious. So did the present model study. We did not perform further study in this paper because of insufficient biological evidence as well as the minor effect. A related study can be conducted if more data are provided.

This simulation study revealed the acute effect of ATO on ionic channel currents rather than on protein expression. In the future, a Markov pharmacological model of *I*_*Kr*_ can be built to simulate the effect of ATO on hERG protein, which can refer to the previous short-QT simulation work (Whittaker et al., 2017; Zhang et al., 2022).

Many drugs or chemical compounds have been explored to relieve ATO toxicity (Haybar et al., 2019), but most of their ionic reactions were not clear, so only two drugs were modeled in this study. Nevertheless, this study provided an approach for simulating and evaluating the effectiveness of new drugs that can ameliorate ATO toxicity. Drugs that can protect the hearts treated with ATO should be compared in the future so that the optimal medication regimen can be screened.

## Conclusion

This study provided a computational method for investigating the cardiotoxicity induced by ATO. The mechanisms of arrhythmia attributed to ATO were investigated from the ionic level to the tissue level. Simulation results showed that ATO not only extended the QT interval of ECG but also aggravated the heterogeneity of VM cells and led to alternans, thus raising the possibility of reentry in the human ventricle. Under the actions of ATO, resveratrol was incorporated into the ventricular model by intervening in the ionic channel, by which the side effects of ATO were ameliorated. The method of this study can also be used to screen drugs that may ameliorate ATO toxicity. This study elucidated ATO cardiotoxicity pathogenesis and its attenuation mechanisms, which is expected to improve ATO treatment in its clinical use.

## Data Availability

The original contributions presented in the study are included in the article/Supplementary Material, further inquiries can be directed to the corresponding authors.

## Supplementary material

Supplementary Figure S1 The calcium dynamic in VMs under the action of ATO. **(A–D)** The membrane potential (*V*), *I*_*CaL*_, *[Ca* ^*2+*^ *]*_*i*_, and *I*_*NaCa*_ of endocardial cells (ENDO) on the condition of control and 3 μM ATO.

Supplementary Figure S2 The action potential of heterogeneous ventricular cable with a BCL of 410 ms.

Supplementary Figure S3 The action potential of endocardial cells (ENDO), middle cells (MCELL) and epicardial cells (EPI) incubated with 3 µM ATO on the condition of pharmacological rescue.

Supplementary Video S1 Control reentry in heterogeneous ventricular tissue. This video shows the generation and elimination of a reentrant excitation wave in a control condition. At the beginning of the simulation, an S1 stimulus was conducted at the peripheral endocardial cells so that a planar wave was initiated. At 378 m, a S2 stimulus was applied at a piece of epicardial cells neighboring the middle cells so that a series of spiral waves were provoked.

Supplementary Video S2 ATO reentry in a heterogeneous ventricular tissue. This video shows the generation and elimination of a reentrant excitation wave in 3 µM ATO. At the beginning of the simulation, an S1 stimulus was conducted at the peripheral endocardial cells so that a planar wave was initiated. At 475 ms, an S2 stimulus was applied at a piece of epicardial cells neighboring the middle cells so that a series of spiral waves were provoked.
