# Supplementary figures and images for: In silico mechanisms of arsenic trioxide-induced cardiotoxicity
Source: Front Physiol. 2022 Dec 15;13:1004605. doi: 10.3389/fphys.2022.1004605 (PMC9798418; doi:10.3389/fphys.2022.1004605)

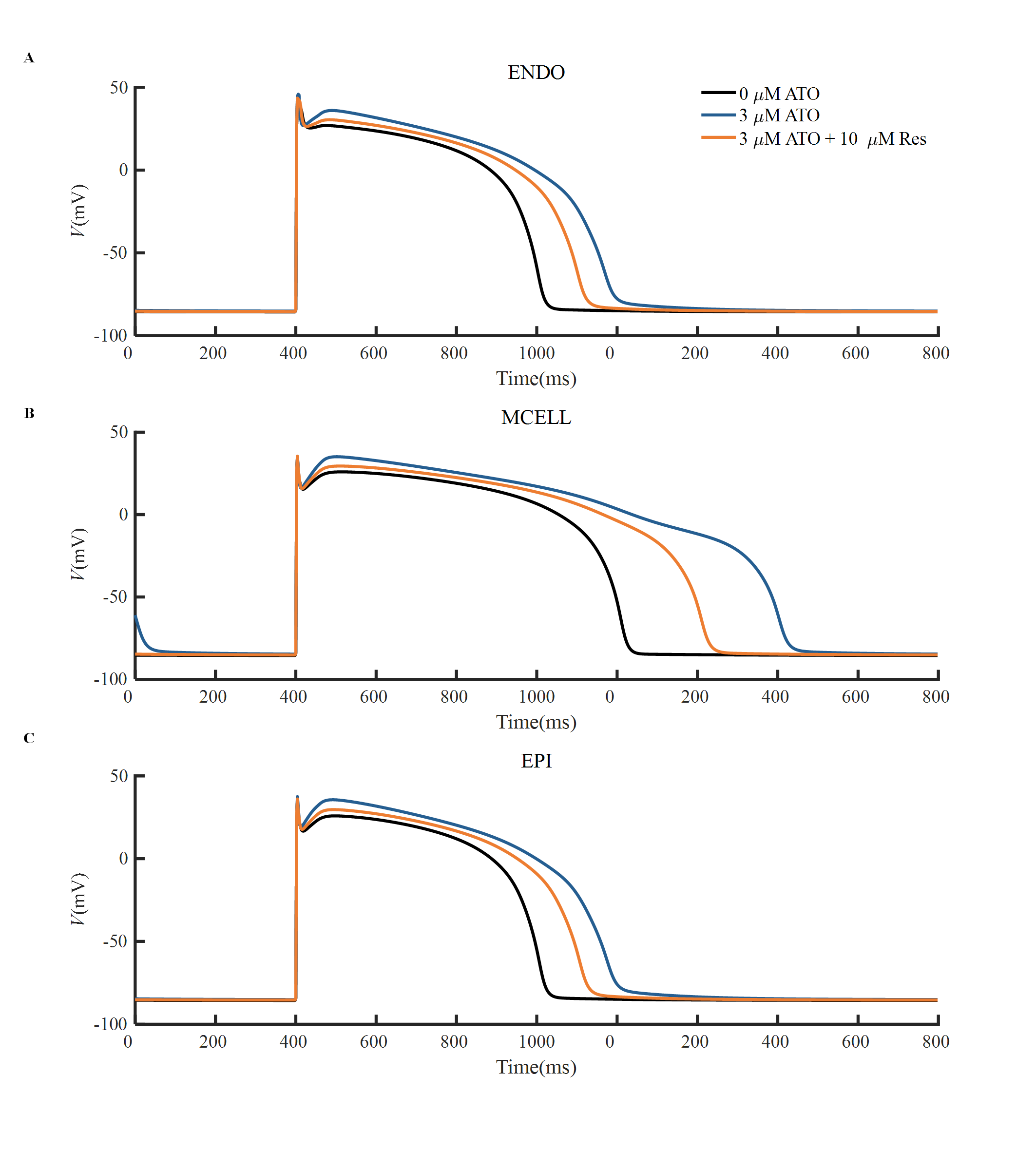

Supplement: Supplementary file 1 [file Image3.TIF]

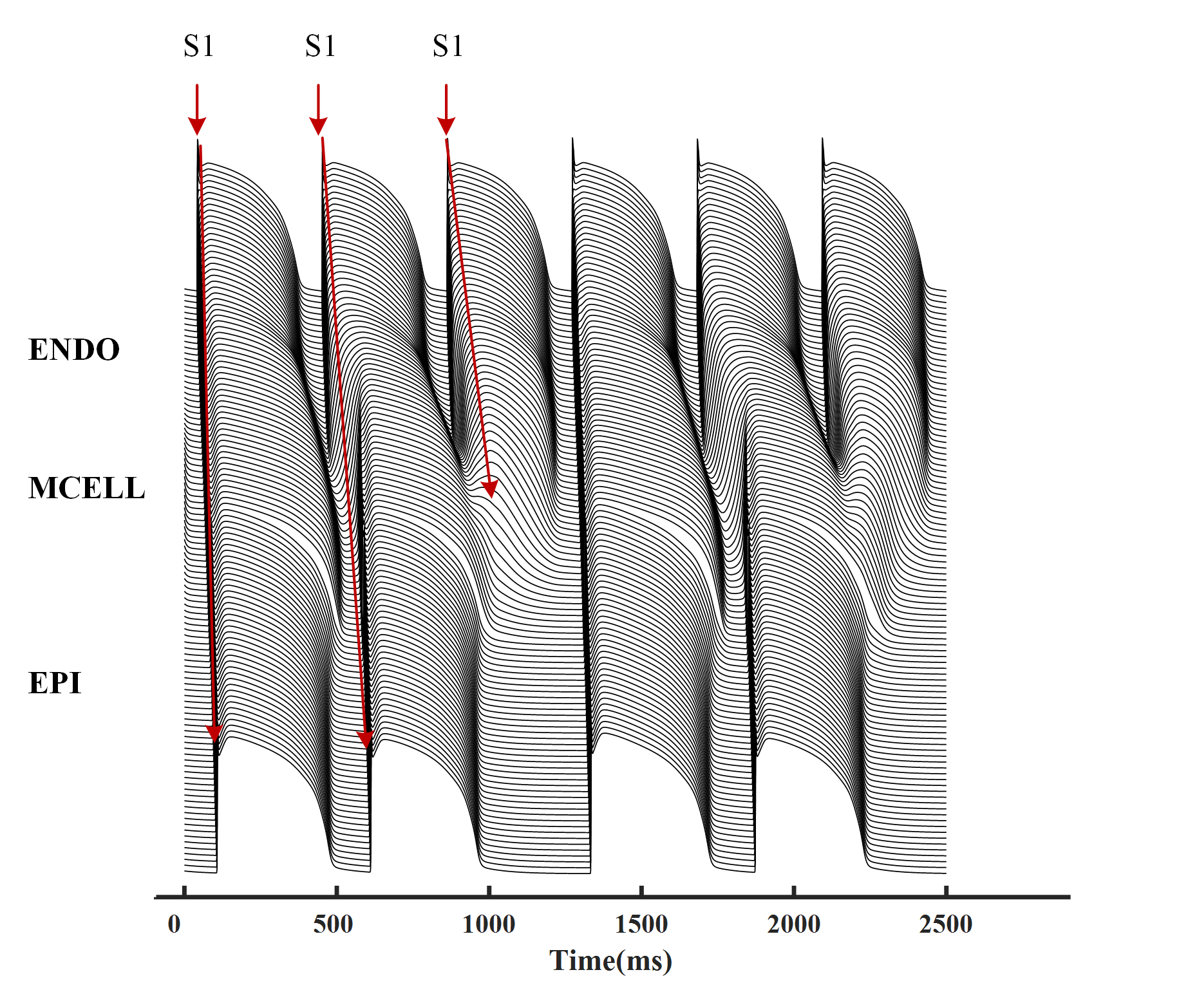

Supplement: Supplementary file 2 [file Image2.TIF]

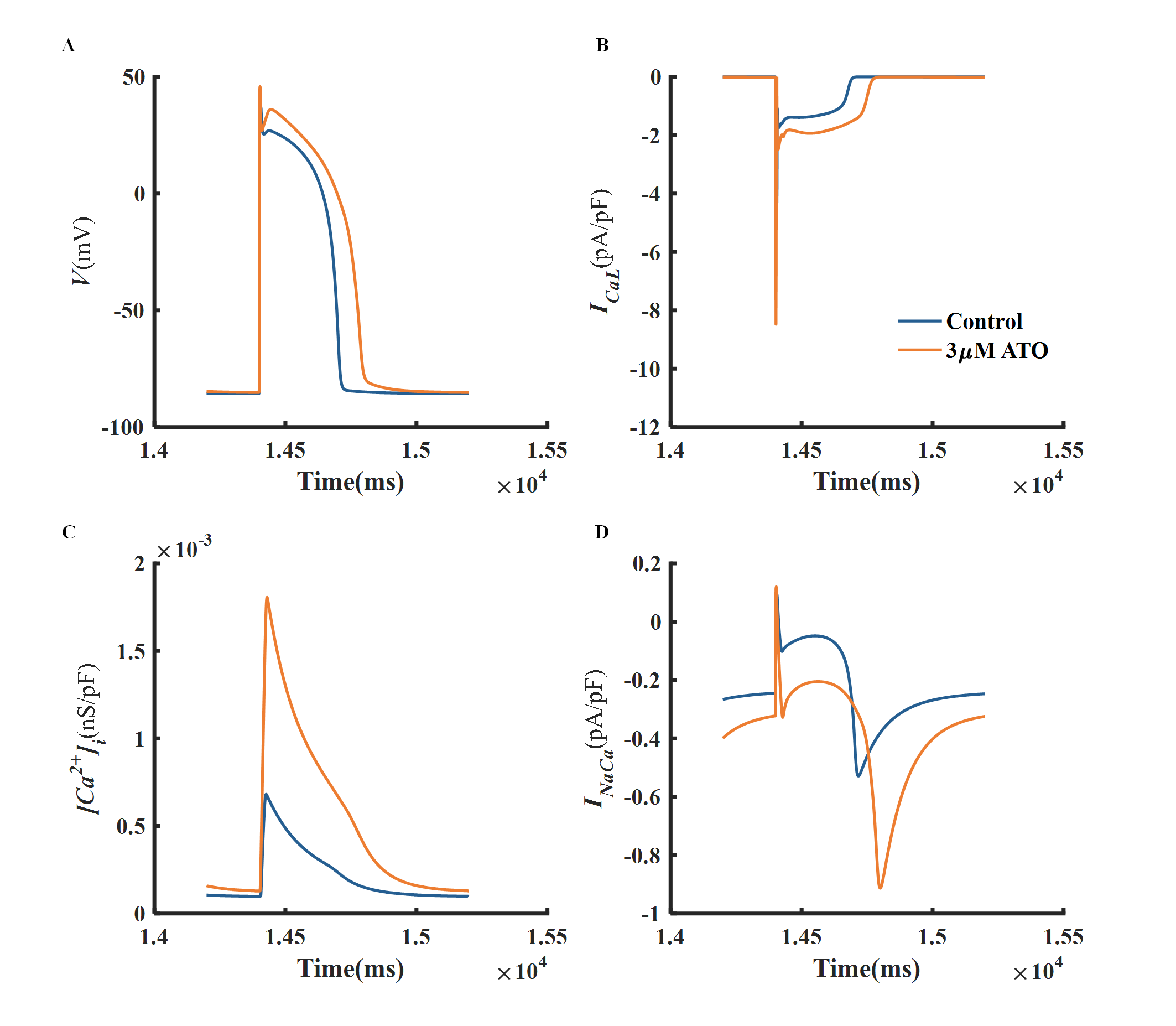

Supplement: Supplementary file 3 [file Image1.TIF]
